# Non-*Leishmania* Parasite in Fatal Visceral Leishmaniasis–like Disease, Brazil

**DOI:** 10.3201/eid2602.191428

**Published:** 2020-02

**Authors:** Malgorzata Anna Domagalska, Jean-Claude Dujardin

**Affiliations:** Institute of Tropical Medicine, Antwerp, Belgium

**Keywords:** Crithidia, Leishmania, visceral leishmaniasis, mixed infection, parasites, Brazil

**To the Editor:** We read with interest the recent article describing involvement of *Crithidia*-related parasites in visceral leishmaniasis (VL) in Brazil (*1*). In 2010, we published a similar study about the identification of *Leptomonas* sp., another monoxenous trypanosomatid, among clinical isolates from VL patients in India: of 120 cultured isolates, 111 were typed as *L. donovani* and 9 as *Leptomonas* sp. (*2*). As in the Brazil study, we infected BALB/c mice with 1 *Leptomonas* isolate; at 45 days postinfection, we found *Leptomonas* and *Leishmania* DNA by PCR in the animals’ spleens. Assuming that sterility was preserved during the experiment, we interpreted that original infection in patients resulted from a mixture of the 2 species, *Leptomonas* overgrew *L. donovani* in culture because of substantial growth advantage of the former, and a few remaining *L. donovani* cells in the culture spread in the animals after inoculation because of their higher in vivo fitness.

We suspect a similar phenomenon could have occurred in Brazil, and additional analyses are required to support the authors’ conclusions. Given that mice were inoculated with clinical isolates, postanimal typing should have been done. Furthermore, linking genotyping information of cultivated (cloned) strains with a patient phenotype is risky because of the selection biases of in vitro isolation and maintenance. Using a recently developed method for direct sequencing of *L. donovani* complex parasites in host tissues, we demonstrated that genotypes of parasites in bone marrow samples differed from derived and cultivated isolates. This result most likely was due to polyclonal *L. donovani* infections and differences in fitness of different genotypes in vitro and in vivo. On the basis of this evidence, we recommend direct parasite sequencing in clinical samples in future work. If impossible, results based on cultured isolates should be interpreted with caution. We recommend a follow-up study to verify the possibility of *Crithidia*/*Leishmania* co-infection and the capacity of *Crithidia* to cause leishmaniasis-like disease as a single infection.
